# Chikungunya virus drives gut microbiota shifts and IFN-Mediated intestinal repair: insights into microbiota-immune interplay

**DOI:** 10.1080/19490976.2025.2512900

**Published:** 2025-06-05

**Authors:** Hongyu Chen, Kaiyun Ding, Cong Tang, Jingwen Xu, Fengyuan Zhang, Yao Yan, Bai Li, Yanan Zhou, Yun Yang, Hao Yang, Qing Huang, Wenhai Yu, Haixuan Wang, Daoju Wu, Shuaiyao Lu, Hongqi Liu

**Affiliations:** a Institute of Medical Biology, Chinese Academy of Medical Sciences and Peking Union Medical College (IMBCAMS&PUMC), Kunming, Yunnan, China; b Yunnan Province Science and Technology Department, Yunnan Key Laboratory of Cross-Border Infectious Disease Control and Prevention and Novel Drug Development, Kunming, Yunnan, China; c National Human Diseases Animal Model Resource Center, NHC Key Laboratory of Human Disease Comparative Medicine, National Center of Technology Innovation for Animal Model, State Key Laboratory of Respiratory Health and Multimorbidity, and Key Laboratory of Pathogen Infection Prevention and Control, Ministry of Education, Institute of Laboratory Animal Sciences, Chinese Academy of Medical Sciences and Peking Union Medical College, Beijing, China

**Keywords:** CHIKV, gut microbiota, interferon, gastrointestinal infection, integrative analysis

## Abstract

Chikungunya virus (CHIKV) infection causes joint damage and gastrointestinal clinical symptoms, including vomiting and diarrhea, particularly in elderly populations, reflecting the potential role of gut immunity in infection. However, the mechanisms by which CHIKV induces gastrointestinal diseases remain largely unexplored. This study investigated the characteristics of fecal and gut microbiota, gut metabolites, and gut immunity post-infection using multi-omics analysis. The role of gut microbiota was further validated through Oral antibiotic depletion (Abx). Importantly, a systematic comparison of age-dependent differences in gut microbiota composition and immune responses following CHIKV infection was conducted to elucidate the involvement of gut microbiota in CHIKV pathogenesis. CHIKV joint inoculation induces gastrointestinal infection and histological damage, drives fluctuations in gut microbiota, markedly increasing the abundance of *Bacteroides fragilis* and *Prevotella sp*. and upregulates conjugates of taurine and bile acids. CHIKV infection further exacerbates systemic inflammatory burden and activates intestinal interferon (IFN) signaling cascades, which supports gut repair and mucosal regeneration, but low antiviral responses to CHIKV infection compared with that of adult animals. Our results suggest that the gastrointestinal tract, along with its microbes and metabolites, modulates CHIKV infection in an age-dependent manner, providing critical insights for diagnosis, treatment, and novel therapeutic development.

## Introduction

Chikungunya virus (CHIKV), an enveloped *Alphavirus* with a single-stranded positive-sense RNA, belongs to the *Togaviridae* family. It has a spherical structure with a diameter of approximately 65 nm. The viral particles comprise an inner layer of capsid protein and an outer layer formed by envelope glycoproteins E1 and E2. The E1-E2 heterodimers trimerize to form viral spikes that mediate receptor binding and membrane fusion, whereas the nucleocapsid encapsulates the viral RNA genome beneath the glycoprotein envelope.^1^

There are four defined CHIKV genotypes: West African, East Central South Africa (ECSA), ECSA-diverged or Indian Ocean lineage, and Asian lineage.^2^ Viral adaptation, climate change, and globalization contributed to the geographical spread of CHIKV, which extends beyond tropical and neotropical regions, with over 100 countries reporting CHIKV transmission. To date, over 10 million cases of chikungunya fever have been reported, and an estimated 1.3 billion individuals worldwide are at risk. Owing to the high pandemic potential of CHIKV, the World Health Organization identified it as a priority for research and development.^3^

CHIKV infection manifests as an acute febrile illness known as chikungunya fever. The infection is self-limiting and characterized by severe polyarthralgia and myalgia (joint and skeletal muscle pain), which can progress to chronic conditions (long-lasting arthralgia) in a substantial proportion of infected individuals and is mostly accompanied by debilitating rheumatic disease.^4^ Gastrointestinal (GI) symptoms, especially abdominal pain and nausea, were reported in up to 66% of CHIKV-infected patients.^5^ However, only two vaccines, Ixchiq and Vimkunya, received the US Food and Drug Administration’s accelerated approval, and no targeted antiviral drugs are available.^6^ The incubation period of CHIKV infection is short (≤3 days), with an acute phase characterized by excessively high viremia levels.^7^ In the acute phase, CHIKV infection targets macrophages and fibroblasts within synovial joints, causing tissue destruction. This drives the production of proinflammatory cytokines and stimulates the influx of immune cells, such as macrophages and T, B, and natural killer cells, to create an inflammatory environment, increasing cytokine levels, such as interleukin (IL)-1β, IL-6, tumor necrosis factor (TNF), monocyte chemoattractant protein-1 (MCP-1; CCL2), interferon-γ (IFN-γ), IFN-α, IL-2, IL-2 R, IL-7, IL-12, IL-15, IL-17, and IL-18.^8^ Furthermore, systemic dissemination of the virus to various organs and tissues following the acute phase underlies the symptoms of chikungunya fever in mouse and non-human primate models.^9^ Notably, epidemiological investigations highlighted the prominent incidence of CHIKV infection in middle-aged and older populations, with fatalities concentrated among older age, suggesting a potential association between CHIKV infection and age distribution.^10^

CHIKV infection-induced chronic joint inflammation is similar to that of rheumatoid arthritis (RA), considering their biomarkers and imaging findings. Additionally, the drugs (DMARDs) for alleviating RA are commonly used to treat chronic joint pain following CHIKV infection.^11^ Approximately 40 Colombian patients with persistent joint pain following initial CHIKV infection showed no detectable viral load 22 months after infection. This suggests the possibility of unclear mechanisms underlying chronic inflammation after CHIKV infection.^12^ The relationship between gut microbiota and arthritis, the “gut-joint axis,”^13^ has been reported. In addition to the classical symptoms of fever, rash, and joint pain, CHIKV infection can also cause a range of other manifestations, including GI symptoms. However, studies of the involvement of the GI tract in the pathogenic mechanism of CHIKV infection have been limited. *Fusobacterium nucleatum* is enriched in patients with RA, positively correlated with serum inflammatory cytokines, and exacerbates RA through FadA-containing outer membrane vesicles.^14^ Boer et al. demonstrated that metabolites from *Streptococcus sp*. may activate synovial macrophages through the gut-blood barrier, triggering systemic inflammatory responses and causing arthritis exacerbations.^15^ Gut microbiota-derived lipopolysaccharides (LPS) are reported to be associated with the severity of knee osteoarthritis and inflammation.^16^ Furthermore, viral infections reportedly drive shifts in the gut microbiota and affect the metabolism of compounds, such as short-chain fatty acids and tryptophan, which indirectly affects host anti-inflammatory and anti-infection responses.^17^ Exogenous supplementation with a butyrate-rich diet in mice promotes joint swelling during CHIKV infection and substantially exacerbates CHIKV-induced arthritis. This underscores the crucial relationship between the gut microbiota and CHIKV infection, particularly in post-infection joint inflammation.^18^ Intriguingly, our prior study demonstrated a potential synergy between CHIKV-induced host immune modulation, arthritic inflammation, and the gut microbiota in a non-human primate model.^19^ However, the inherent limitations of non-human primate models constrain further mechanistic investigations, leaving the findings primarily descriptive. In this study, we leveraged a C57BL/6 mouse model of CHIKV infection to i) comprehensively characterize the features of infection across different host ages, employing multi-omics approaches, including metagenomics, transcriptomics, and metabolomics, complemented by experimental validations. ii) Critically, we utilized antibiotic-mediated depletion of gut microbiota to elucidate its role in host immune regulation during CHIKV infection. This study enhances the understanding of the role of the gut environment in the pathogenesis of CHIKV infection. It also provides new insights into the mechanisms underlying CHIKV-induced arthritis.

## Results

### CHIKV joint inoculation causes GI infection and histological damage

**Figure 1.**
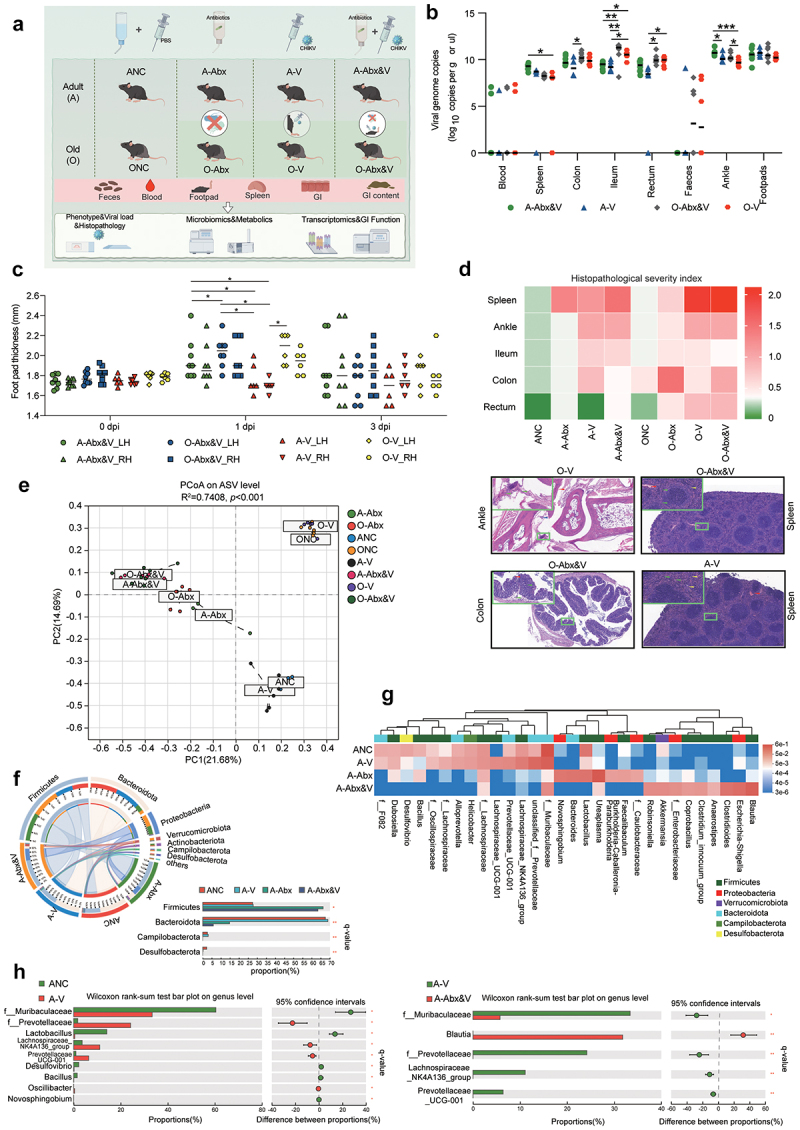
Histopathological analysis and characteristics of the microbiome in feces of chikungunya virus (CHIKV)-infected mice. Animal experiment was done as outlined in the following scheme. (a) Scheme of this study. adult (a) or elderly (O) mice (C57BL/6) were randomized into nontreatment control. The animals were infected with CHIKV at 2 × 10^7^ plaque-forming units (PFU) via leg muscle injection. Fresh fecal samples were collected during the acute phase. On day 3 post infection, all animals were euthanized and dissected for collection of GI content and tissue samples, followed by evaluations of viral load, pathological histology, transcriptomics, *16S* rRNA sequencing, microbial metagenomics, and metabolomics. This figure was prepared using the online software FigDraw (copyright no. SPSAW79686). (b) Viral genomic RNA was detected via Taq-man RT-PCR in the blood, spleen, gastrointestinal (GI) tissues, ankles, footpads, and feces of CHIKV-infected mice. The details are described in the section methods. Statistical difference analysis was examined (one-way ANOVA and post hoc *Scheffe* test, FDR corrected; *q < 0.05, **q < 0.01, ***q < 0.005, and ****q < 0.001). (c) Mouse footpad thickness was compared among the indicated groups (L, left footpad; R: right footpad). Statistical difference analysis was examined (one-way ANOVA and post hoc *Scheffe* test, FDR corrected; *q < 0.05). (d) Histopathological severity index (top panel) was plotted as a heatmap via the software Graphpad on basis of scores of histological lesion that is described in the section methods. The representative histological images were shown (bottom panel). (e) Principal coordinate analysis (PCoA) is performed for the microorganisms in the feces of the adult groups based on ASV levels post *16S* rRNA sequencing. Statistical differences among clusters were analyzed using permutational analysis of variance (PERMMANOVA). R^2^ was used to interpret the degree of difference among each group. Statistical significance was set at *p*<0.05. (f) Composition of the fecal microbiota at the phylum level in adult groups is plotted using circos-0.67–7 (http://circus.Ca/). Ribbons of the left half-circle represent the composition (outside) and proportion (inside) of microbiota phyla, respectively. Ribbons of the right half-circle represent the distribution proportion (outside) and the relative abundance (inside) of the microbiota phylum in each group, respectively. (g) The composition of the fecal microbiota at the genus level in adult groups was plotted as a heatmap (data were normalized by Z-score standardization method). Colored bars on the top of heatmap represent the microbiota phyla. The microorganisms that can not be annotated to the genus level were re-annotated to the family level with the labeling prefix “f__”. (h) Comparisons of fecal microbiota proportion between the ANC and A-V groups (left), and between A-V and A-Abx&V groups (right). Fecal microbiota at the top 30 proportion (genus-level) were chosen for further statistical analysis. Microbiota with significant differences were shown here. (*Wilcoxon rank-sum test and* post hoc *scheffe test*, FDR corrected; *q < 0.01, **q < 0.001). The microorganisms that can not be annotated to the genus level were re-annotated to the family level with the labeling prefix “f__”.

A CHIKV mouse model was used to investigate how alterations in the gut environment influence disease outcomes, as well as GI and gut microbiota responses to viral infection (Figure 1(a)). After CHIKV joint infection, viral genomic RNA was detected in the blood, spleen, ankle, and footpad samples of all infected animals, with varying degrees of footpad swelling and histological lesions (Figure 1(b-d)). Notably, viral genomic RNA and histological lesions (inflammatory cell infiltration, epithelial detachment, mucosal edema, and cell necrosis) were also observed in the ileum, colon, rectum, and feces of CHIKV-infected animals (Figure 1(b,d,) Supplementary Figure S1A). The elderly group showed higher numbers of viral genomic RNA and more severe pathological and histological lesion than those of the adult group, suggesting that the severity of CHIKV infection may be age-related and can be detoxified through the digestive tract. To further confirm the effects of GI microbes on CHIKV infection, microbiota depletion by multiple antibiotics treatment was conducted before the viral infection. Antibiotic treatment led to higher viral load and more severe histopathology in GI tissues than that with CHIKV. However, antibiotic treatment was not the primary cause of the pathologic damage (Figure 1(d)).

To determine the specific species of microorganism that are associated with CHIKV infection, we performed *16S* rRNA sequencing on fecal microbiota during the acute phase of viremia and incorporated microbiomes affected by antibiotic consumption to further validate the role of microbes following infection. Beta diversity (principal coordinate analysis, PCoA) of the fecal microbiota revealed no significant clustering between ANC and A-V, or between ONC and O-V, either at the PC1 or PC2 level. Notably, there were differences in fecal microbes (composition and structure) between the ONC and ANC groups. However, infection after antibiotic-microbial depletion significantly altered the fecal microbiota composition, leading to the formation of independent sub-clusters and causing convergence in the fecal microbiota of the A-Abx-V and O-Abx-V groups (Figure 1(e)). Furthermore, there was no significant difference in the Alpha diversity index of the fecal microbiota between the CHIKV infection group (A-V) and the NC group (Supplementary Figure S1B). At the phylum level, CHIKV infection significantly reduced the Desulfobacterota phylum. Moreover, the abundance of the Bacteroidota phylum was significantly reduced by antibiotics and further decreased after CHIKV infection (Figure 1(f)). Analysis of microbiota composition revealed that the abundances of *Muribaculaceae*, *Lactobacillus*, and *Bacillus* genera were significantly decreased post-CHIKV infection (A-V vs non-treatment control [ANC]). However, the abundances of *Prevotella*, *Prevotellaceae-UCG001*, and *NK4A136* were significantly increased post-CHIKV infection (A-V vs ANC) (Figure 1(g,h)). The shifts of fecal microbiota caused by CHIKV infection were similar between the elderly and adult groups. Notably, although elderly antibiotic-treated mice (O-Abx) exhibited stronger fecal microbiota perturbations compared to adult counterparts (A-Abx), CHIKV infection induced additional divergence beyond antibiotic-driven alterations. Strikingly, this age-independent convergence in gut microbial composition and beta diversity (Figure 1(e)) implies CHIKV-mediated homogenization of intestinal ecosystems. Importantly, such microbiota destabilization may potentiate CHIKV pathogenicity by exacerbating host-microbiota-virus interactions.

### CHIKV infection drives gut microbiota fluctuations and upregulates bile acid levels

**Figure 2.**
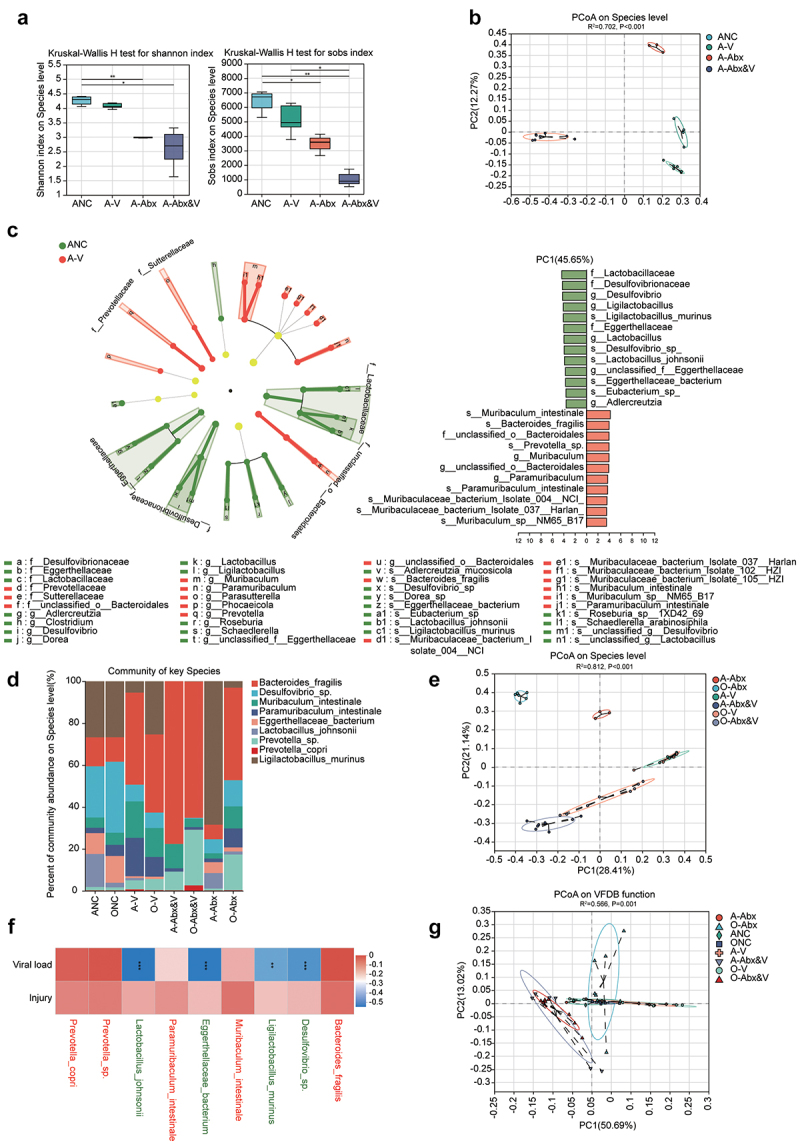
Alterations of gastrointestinal microbiome in CHIKV-infected mice. GI contents of animals were harvested for metagenomic sequencing at the indicated time point as described in Figure 1(a). Raw data was processed as described in methods. (a) The alpha diversity of the gut microbiota was calculated, followed by examination of statistical difference among groups (*Wilcoxon rank-sum test* and post hoc *Scheffe* test, FDR corrected; *q < 0.05, **q < 0.01) (left panel, Shannon indices; right panel, sobs indices). (b) PCoA of microorganisms in the GI contents of the adult group was performed at the species level. Statistical differences among clusters were analyzed via PERMANOVA. R^2^ was used to interpret the degree of difference among the groups. Statistical significance was set at *p* < 0.05. (c) LEfSe was conducted to identify the key microbes at the species level contributing significantly to the differences among groups after CHIKV infection (LDA score ≥ 4). The microorganism that can not be annotated to the genus level was re-annotated to the family level with the labeling prefix “f__”. (d) The composition and structure of the key microbes (identified by LEfSe) contributing significantly to the differences among groups were determined as described in the section of methods. (e) PCoA was conducted to analyze the microorganisms at the species level in the GI contents of the infected groups (adult and elderly). Significant differences among clusters were determined using PERMANOVA. R^2^ was used to interpret the degree of difference between the groups. Statistical significance was set at *p* < 0.05. (f) The correlation matrix shows relations between the crucial microbes and high viral load or pathological damage. The levels of correlation (Spearman correlation) are proportional to color density, as shown in the scale bar. Significance is marked with * (**q < 0.01, and *** q < 0.005). Microbe species in red and green letters represents the increase and decrease of microbial abundance post CHIKV infection, respectively. (g) PCoA was performed to analyze the microorganisms in the GI contents of all groups on basis of the functions of virulence factor. Significant differences among clusters were analyzed using PERMANOVA. R^2^ was used to interpret the degree of difference among the groups. Statistical significance was set at *p* < 0.05.

The fecal microbiota analysis above suggests that CHIKV infection induces gut microbial alterations. However, fecal microbiota may not accurately reflect the full spectrum of microbial diversity within the entire gut.^20^ Metagenomic sequencing was conducted to determine microorganisms in the intestinal contents of CHIKV-infected mice and to precisely characterize the GI microbiome post-CHIKV infection. CHIKV infection caused a decrease in diversity and richness of GI microbiota (Figure 2(a,b)). Linear discriminant analysis effect size (LEfSe) analysis further revealed that the main microbiota affected by CHIKV infection were *Bacteroides fragilis*, *Ligilactobacillus murinus*, *Muribaculum intestinale*, *Paramuribaculum intestinale*, and *Prevotella sp*. (Figure 2(c,d)). Additionally, the GI microbiota of the adult and elderly groups showed convergence post-CHIKV infection, whereas antibiotics treatment caused obvious clusters of GI microbiota between the two age groups (A-Abx and O-Abx). However, GI microbiota was re-convergenced by the pressure of CHIKV infection (Figure 2(e)).

Furthermore, abundance analysis of the GI microbiota showed that shifts in response to CHIKV infection were greater in the elderly group than in the adult group, although their shift tendencies were similar (Supplementary Figure S2A). *B. fragilis*, *M. intestinal*e, and *P. intestinale* showed significantly increased abundances in CHIKV-infected adult animals (*p* < 0.05). In contrast, *Desulfovibrio sp*., *Eggerthellaceae bacterium*, and *L. murinus* decreased in infected adult groups. Similar trends were observed in the group O-Abx&V. *B. fragilis* significantly decreased with antibiotics treatment in the adult group were recovered by CHIKV infection (A-Abx-V), the effect of which is attenuated in the elderly groups. Notably, antibiotic depletion exacerbated the impact of CHIKV infection on GI microbiota by increasing the abundances of *B. fragilis*, *Prevotella sp*., *Desulfovibrio sp*., and reducing *E. bacterium*. Correlation matrices confirmed positive associations between these key microbiota, increased abundance post-infection, high GI viral loads, and tissue damage (Figure 2(f)). Furthermore, the abundance of the microbiota related to boosting immune health functions decreased after CHIKV infection (Supplementary Fig. S2B), similar to that with probiotics, particularly in the elderly CHIKV group (O-V) (Supplementary Fig. S2C). Similarly, microbes involved in the digestive system functions exhibited decreased abundances (Supplementary Fig. S2D). *Bacteroidales bacterium*, *M. intestinale*, *P. intestinale*, and *Prevotella sp*., which exhibited increased abundances post-CHIKV infection, significantly contributed to functions of the glycosyltransferase family GT2, GT4, GT41 (Des), and GH2 enzymes (Supplementary Fig. S2E). Function annotation post PCoA of the virulence factors of pathogenic bacteria (Virulence Factor Database, VFDB) further confirmed the functional shifts caused by CHIKV infection, particularly in the Abx-infected groups (A-Abx-V and O-Abx-V) (Figure 2(g)). *B. fragilis* and *Prevotella sp*., which showed increased abundances post-infection, significantly contributed to the expression of various virulence factors (Supplementary Fig. S2F). These results demonstrate that CHIKV infection drives shifts in the GI microbiota and converges between the adult and elderly groups, including increased abundances of *B. fragilis*, *M. intestinale*, and *P. intestinale*. These shifts enhance the functions of microbial virulence factors and suppress probiotic abundance. Depletion of the GI microbiota with antibiotics followed by CHIKV infection further amplified these shifts, particularly for *B. fragilis* and *Prevotella sp*. We hypothesized that these changes (microbiota alternation) might contribute to increased viral loads and tissue damage caused by CHIKV infection. Although the crucial microbial changes in the adult and elderly groups had similar trends, the elderly group had lower sensitivity.

**Figure 3.**
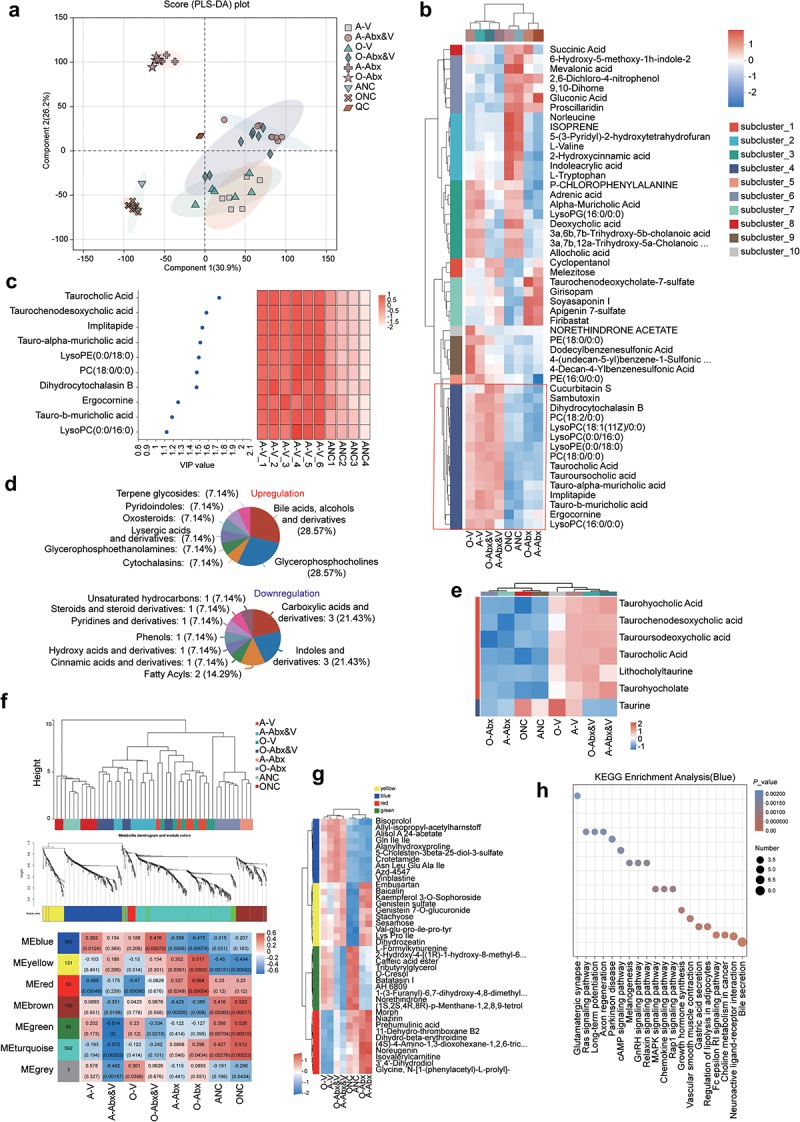
Distinct profiles of GI metabolites in mice post-CHIKV infection. GI contents of animals were harvested for non-targeted metabolomics analysis at the indicated time point as described in Figure 1(a). Raw data was processed as described in methods. (a) PLS-DA was performed and plotted for metabolites in the GI contents. (b) The metabolites at the top 50 abundance in the GI contents were plotted as a heatmap. The hierarchical clustering (left rainbow bar) was determined according to metabolite levels. Metabolites increased in all groups with CHIKV infection are shown in red box. (c) The metabolites at the top ten abundances contributing to CHIKV infection were chosen for variable importance (VIP) analysis (left panel), which was plotted as a heatmap (right panel, data were normalized by Z-score standardization method). (d) Functional enrichment analysis was performed for the upregulated (top) and downregulated (bottom) metabolites by CHIKV infection. (e) Taurine and bile acid-conjugated metabolites were plotted as a heatmap on basis of their abundences (data were normalized by Z-score standardization method). (f) WGCNA was performed to identify hub metabolites and their correlations with each group. Top panel, sample clustering dendrogram. Middle panel, metabolite clustering dendrogram. Bottom panel, correlation of modules with groups, in which the number in the left column (modules) indicates the number of metabolites in each module. The numbers in the heatmap indicate the correlation coefficients and *p* values (in parentheses) between the modules and the corresponding group (red means positive correlation, and blue means negative correlation). (g) Metabolites at the top 10 abundance in the blue, yellow, green and red modules from fig 3f were plotted as a heatmap (data were normalized by Z-score standardization method). (h) KEGG functional enrichment analysis of metabolites in the blue module from WGCNA in Fig 3f was performed as described in the section of methods. The size of the circle represents the amount of metabolites enriched in the indicated function.

To further understand the impact of CHIKV infection on the digestive tract, a non-targeted metabolomic analysis of microbiota in the intestinal contents was performed. CHIKV infection altered the composition and structure of GI metabolites. Furthermore, the GI metabolite structures were more similar between the Abx (A-Abx-V) and direct infection groups (A-V) (Figure 3(a)), suggesting that Abx treatment was not the primary factor affecting the GI metabolites. Metabolites in sub-cluster 4 increased after CHIKV infection, particularly in the Abx-infected groups (O-Abx-V and A-Abx-V) (Figure 3(b)). Variable importance analysis (VIP) was conducted to identify the upregulated metabolites following CHIKV infection. Taurocholic, taurochenodeoxycholic (TCDCA), and tauroursodeoxycholic acids were upregulated (Figure 3(c)), which belong to primary bile acids, alcohols, and derivatives (Figure 3d). Notably, taurine levels increased post-infection, but were suppressed by antibiotics, which might explain its reduced levels in the Abx-infected group (Figure 3(e)). Weighted gene co-expression network analysis (WGCNA) further validated the modules associated with CHIKV infection (Blue) (Figure 3(f,g)), which were also significantly enriched in bile secretion-related functions (Figure 3(h)). However, other bile acid metabolites decreased post viral infection (Supplementary Fig. S3A). There was positive associations between the vital microbiota with increased abundances post-infection (*B. fragilis*, *Prevotella sp*., and *M. intestinale*) and conjugates of taurine and bile acids (Supplementary Fig. S3B). These results demonstrate that CHIKV infection affects the composition of intestinal metabolites, particularly in the Abx-infected groups and upregulates the bile acid levels, particularly taurocholic and tauroursodeoxycholic acids, in the gut.

### CHIKV infection promotes systemic inflammatory burden and activates intestinal IFN signaling to assist gut repair and mucosal regeneration

**Figure 4.**
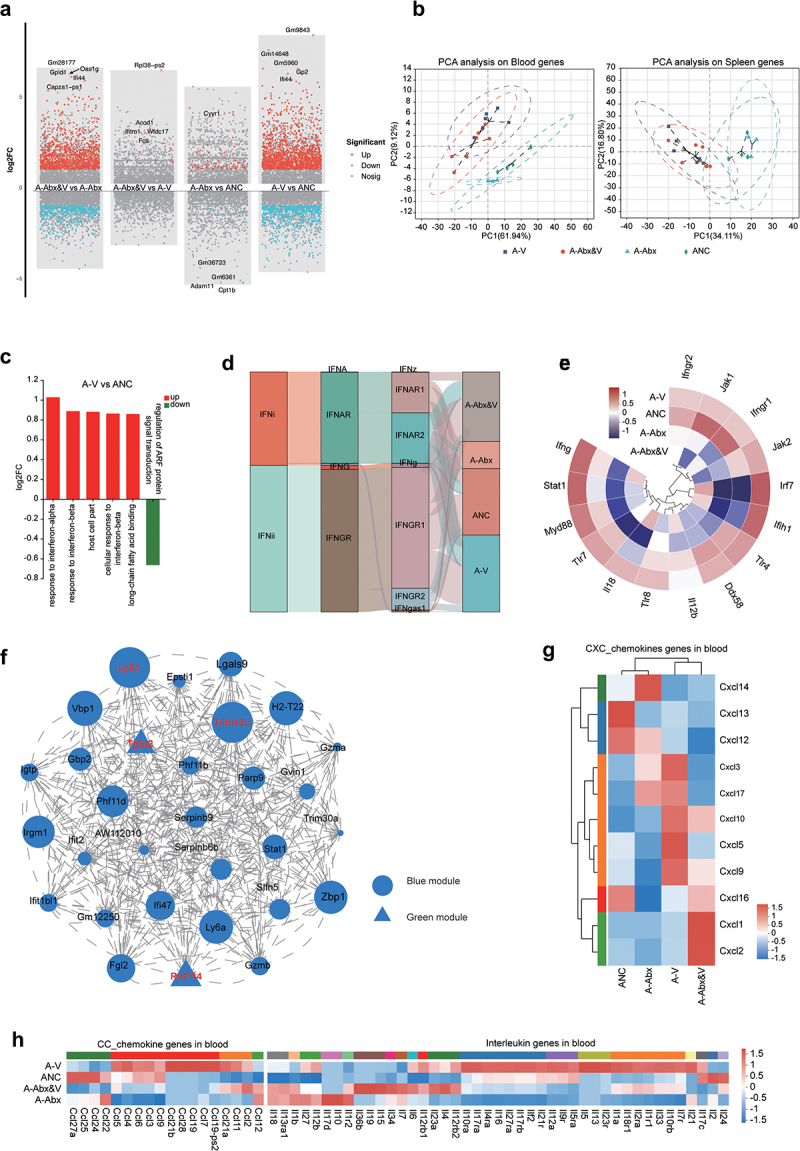
Transcriptomic characteristics of the blood and spleen. Blood and spleen of animals were collected for transcriptomic analysis at the indicated time point as described in fig 1a. Raw data was processed as described in methods. (a) Number of DEGs in the blood of adult groups was plotted as a dot graph, in which each dot represents one gene. (b) PCA of gene expression in the blood (top) and spleen (bottom) of the adult groups was performed and plotted. (c) Functional enrichment analysis was conducted for significantly upregulated and downregulated DEGs in blood from adult groups (A-V vs ANC). (d) Abundance of interferons (IFNs) and IFN receptor genes in the blood of adult groups was plotted as a Sankey diagram. (e) The relative expression of IFN and interferon-stimulated genes (ISGs) in the blood of adult groups were plotted as a radar heatmap (data were normalized by Z-score standardization method). (f) The top 30 hub genes in the module that showed significantly positive correlation with CHIKV infection were revealed by WGCNA. Each node represents a gene and node size is proportional to connectivity. Hub genes in red letters are related with Supplementary fig. 3e. Expression levels of CXC chemokine genes (g), CC chemokine genes and interleukin genes (h) in blood samples of adult groups were displayed and compared in a heatmap (data were normalized by Z-score standardization method).

To understand host immune regulation post-CHIKV infection and the immune involvement of the GI tract, RNA-seq was performed on whole blood, spleen, and GI tissues of the host. The results illustrated that CHIKV infection caused the increase of differentially expressed gene (DEG) in the blood and spleen, whereas antibiotic treatment caused limited DEG elevation (Figure 4(a), Supplementary Fig. S3C). PCA validated the impact of CHIKV infection on gene expression in the blood and spleen (Figure 4(b)). Gene Set Variation Analysis showed that the upregulated genes after CHIKV infection are primarily related with responses to blood IFN-α and IFN-β (significantly enriched) (Figure 4(c)). Similarly, spleen DEGs were significantly enriched in response to IFN-β (Supplementary Fig. S3D). The Sankey diagram of IFN and receptor genes showed that Type I interferon (*IFN-α*) and its receptor genes (*IFNAR1* and *IFNAR2*), Type II interferon (*IFN-γ*) and its receptor gene (*IFNGR*) were upregulated post-infection (Figure 4(d)). Moreover, IFN pathway-related genes, *TLR4*, *TLR7*, and interferon-stimulated factors (*IL1*8, *ifih1*, *lrf7*, *Myd88*, and *stat1*), were upregulated post-infection. Antibiotic treatment suppressed the expression of IFNs and their associated genes, which were reactivated by CHIKV infection (Figure 4(e)). Furthermore, WGCNA revealed that the blood modules were positively correlated with CHIKV infection (Supplementary Fig. S3E) and primarily enriched in the positive regulation of the IFN-γ response (Supplementary Fig. S3F). Network connectivity analysis further revealed the localized hub genes, *Ly6C*, *Trim12c*, *Tgtp2*, and *Rnf114* (Figure 4(f)). CHIKV infection significantly promoted small intestinal Ly6C expression, particularly in the adult CHIKV group. However, microbial depletion caused a downregulated expression of Ly6C (compared with that of the infected group). In the large intestine, *Ly6C* expression was higher in the elderly group (ONC) compared with that in the adult group (ANC, A-Abx) (not significant) (Supplementary Fig. S3G).

**Figure 5.**
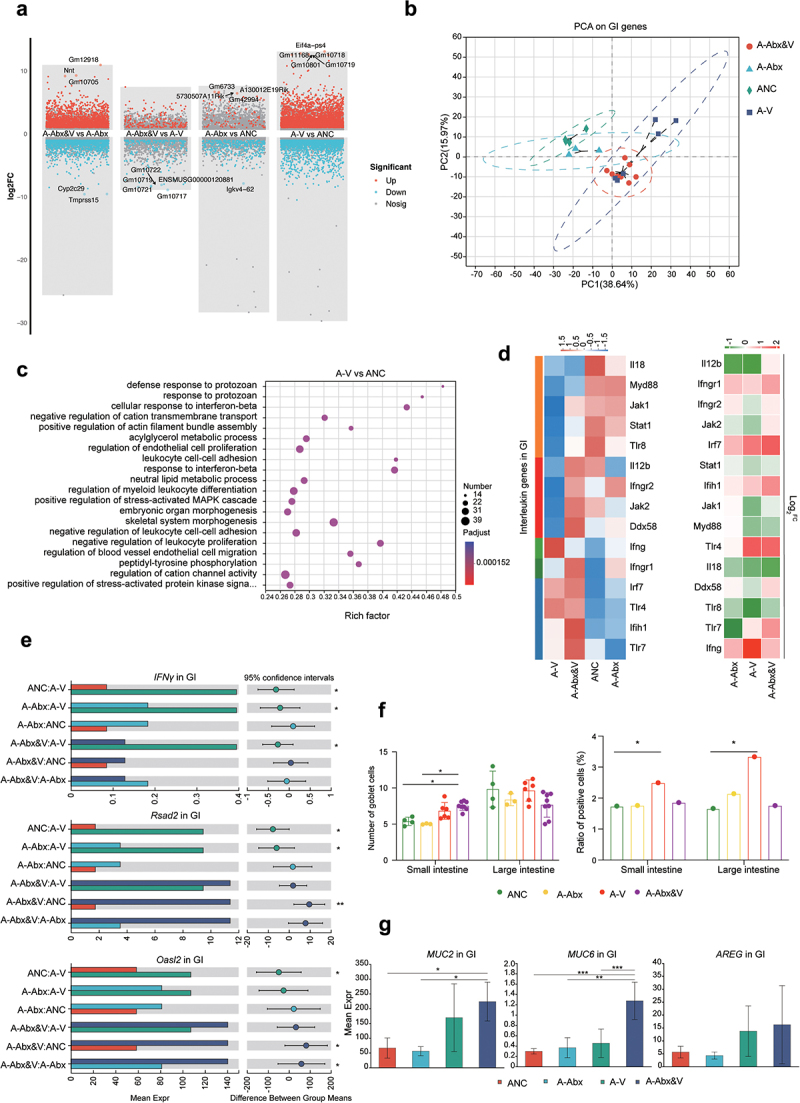
Effects of antibiotic treatment and CHIKV infection on expression of intestinal integrity-associated genes. GI tissues of animals in adult groups were collected for following analyses at the indicated time point as described in fig 1a. (a) Number of DEGs in the blood of adult groups was plotted as a dot graph, in which each dot represents one gene. (b) PCA of gene expression in the blood (top) and spleen (bottom) of the adult groups was performed and plotted. (c) Functional enrichment analysis was performed for DEGs in the intestine (A-V vs. ANC). The size of the circle is proportional to the number of DEGs enriched for in adult groups following CHIKV infection. The *p*-values (two-sided hypergeometric test) are expressed on basis of color density, as shown in the scale bar. (d) Expressions of *IFN* and *ISG* genes in GI tissues were plotted as heatmaps on basis of expression level (left panel) and the log2 Fold change (vs. NC) of the mean value of differentially expressed level (right panel). (e) Statistical difference was examined for gene expression levels of *IFNγ* (top), *Rsad2* (middle), and *Oasl2* (bottom) among groups (*Wilcoxon rank-sum test* and post hoc *scheffe test*, FDR corrected; *q < 0.05, **q < 0.01). (f) Mucosal barrier integrity of the GI tract was evaluated post Alcian blue and periodic acid-schiff (AB-PAS) staining as described in the methods section (left panel) (*one-way ANOVA and* post hoc *scheffe test*, FDR corrected; *p < 0.05). The representative images from AB-PAS staining are shown in Supplementary fig. 1C. Apoptosis and proliferation of cells in the indicated tissues were revealed by immunohistochemical (IHC) staining with the markers, cleaved caspase-3 and KI-67 (right panel). The ratio of the staining density was plotted and statistically analyzed (*one-way ANOVA* and post hoc *scheffe test*, FDR corrected;*q < 0.05). The representative images from IHC staining are shown in Supplementary fig. S4A. (g) Statistical comparisons of gene expression of *MUC2* (left), *MUC6* (middle), and *AREG* (right) in the GI tissues among the indicated groups (*Wilcoxon rank-sum test* and post hoc *scheffe test*; FDR corrected; *q < 0.05, ** q < 0.01, ***q < 0.005).

**Figure 6.**
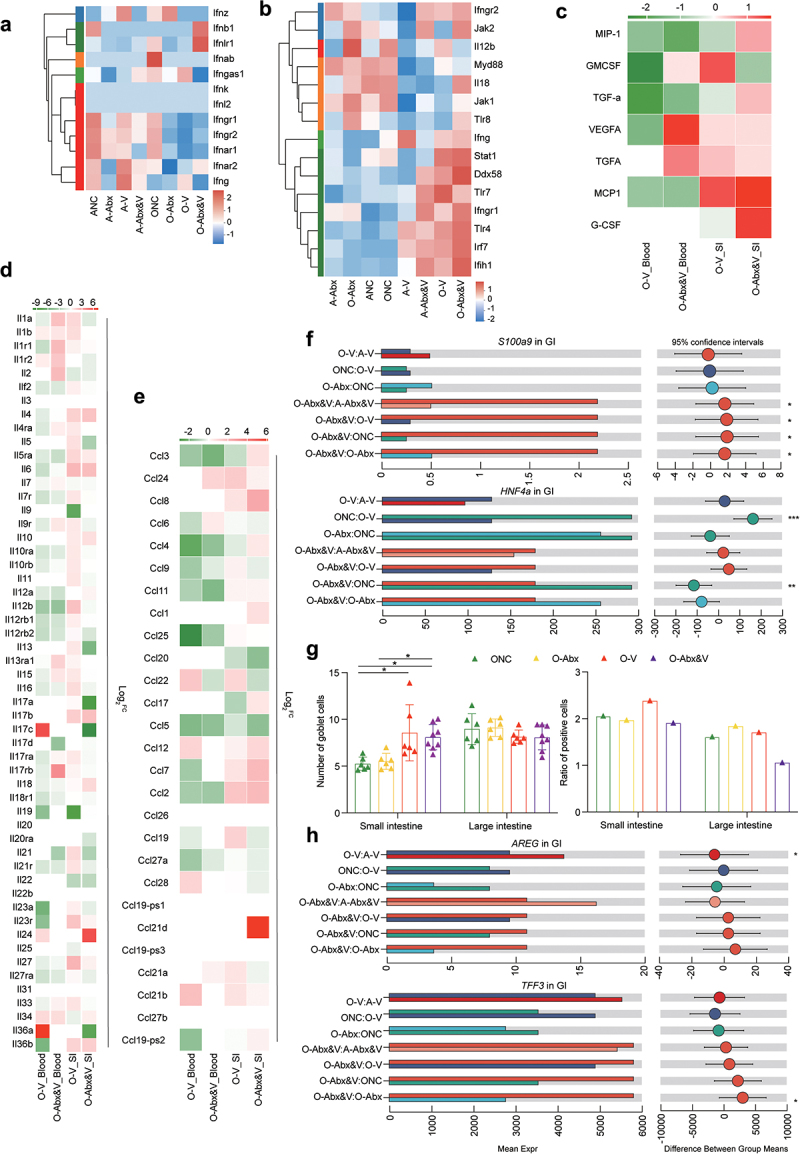
Gene expression profiles in the blood and intestine of the elderly groups reflect a pronounced systemic inflammatory burden post CHIKV infection. Blood and GI tissues of animals in the elderly groups were collected for transcriptomic sequencing, followed by analysis of DEGs. Expressions of IFN and IFN receptor genes in the blood of all groups (a) and ISGs genes in the GI tract of all groups (b), inflammatory cytokine genes in the GI tract and blood of all infected groups (c) were plotted as heatmaps (data were normalized by Z-score standardization method). The log2 Fold change (vs. ANC and ONC) of the mean differentially expressed levels of interleukin genes (d) and chemokine ccl genes (e) was calculated and plotted by the software Graphpad. (f) The gene expression levels of *S100a9* (top) and *HNF4a* (bottom) were calculated by R (version 3.3.1) stats package (*Wilcoxon rank-sum test* and post hoc *scheffe test*; FDR corrected; *q < 0.05, ** q < 0.01). (g) Mucosal barrier integrity of the GI tract from mice in the elderly groups was evaluated using AB-PAS staining (left panel), as described in the section methods. Compared with the NC group, a significant difference of the goblet cell number in the GI tissues of infected animals is indicated by the *one-way ANOVA* and plotted using GraphPad Prism 8.0. *(*p < 0.05). The representative images from AB-PAS staining are shown in Supplementary fig. 1c. Apoptosis and proliferation of cells in the indicated tissue samples were evaluated via IHC staining for the markers, cleaved caspase-3 and KI-67 (right panel). The ratio of the staining density was plotted and statistically analyzed (*one-way ANOVA* and post hoc *scheffe test*, FDR corrected;*q < 0.05). The representative images from IHC staining are shown in Supplementary fig. 4A. (h) The gene expression levels of *AREG* (top) and *TFF3* (bottom) in the GI tract were compared among the groups (*Wilcoxon rank-sum test* and post hoc *scheffe test*; FDR corrected; *q < 0.05, **q < 0.01).

To comprehensively understand systemic immune responses to CHIKV infection, cytokine and chemokine gene expression in the blood were analyzed. CHIKV infection led to the upregulation of multiple chemokines (*CXCL5*, *CXCL3*, and *CXCL9*), substantial upregulation of address in genes, *IL1a*, *IL15*, and *IL18r* in the blood (Figure 4(g,h)), and similarly in the GI tract (Figure 5(a,b)), which was primarily driven by infection rather than antibiotic treatment. DEGs significantly enriched in the GI were also related to the responses to IFN-α and IFN-β (Figure 5(c)). Compared with those in the NC group, *TLR4*, *TLR7*, *IRF7*, *IFIH1*, and *IFNGR* expressions were significantly upregulated in the CHIKV groups, particularly the Abx-CHIKV group (A-Abx-V) (Figure 5(d)). Additionally, the IFN-stimulated genes (ISGs), *Rsad2* and *Oasl2*, were significantly upregulated in the GI tract post-CHIKV infection, particularly in the Abx-CHIKV group. *IFNγ* was significantly upregulated in the GI of the adult CHIKV group (Figure 5(e)). IFN signaling promotes the regeneration of intestinal mucosal epithelial cells. Therefore, the GI barrier was evaluated via Alcian blue and periodic acid – Schiff (AB-PAS) staining of mucosal goblet cells post-CHIKV infection. The results illustrated an increased proportion of goblet cells in the small intestine post-infection (significantly higher in A-Abx-V), which was unrelated to the antibiotic treatment (Figure 5(f)). Consistently, mucin genes, *MUC2* and *MUC6*, were highly expressed in the intestines of adult CHIKV infection groups, significantly in the adult Abx&V group (Figure 5(g)). The recovery marker of mucosal barrier, amphiregulin (*AREG*), was also upregulated in the intestine of A-V and A-Abx&V. The ratio of Ki67 to cleaved caspase-3 (CC3) significantly increased in the adult CHIKV group (Figure 5(f), Supplementary Fig. S4A). No significant differences was observed in zonula occludes protein 1 (ZO-1) and epithelial cell adhesion molecule (EpCAM) expression between non-infection and CHIKV infection groups, suggesting that CHIKV infection caused no mechanical damage to the GI tract (Supplementary Fig. S4B). Significant upregulations of IFNs and their receptors were observed in the GI tract, but not in blood of the elderly group (particularly in O-Abx&V). Additionally, genes for inflammatory cytokines (Figure 6(c)), ILs (Figure 6(d)), and chemokines (Figure 6(e)) were highly expressed in the GI tract of elderly groups, particularly in O-Abx&V (compared with those in adult groups). CHIKV infection led to infiltration of CD4^+^ cells in the small and large intestines (significantly in the large intestine of O-Abx&V) (Supplementary Fig. S4C). Furthermore, the increase of the inflammation marker gene *S100a9* expression confirmed strong intestinal inflammation in the elderly group (particularly O-Abx&V) (Figure 6(f)). *HNF4a* was significantly downregulated in the elderly group, further verifying the strong disturbance of CHIKV infection on GI homeostasis (Figure 6(f)). CHIKV infection caused no mechanical damage in the GI of adult and elderly groups (Supplementary Fig. S4). Goblet cells in the small intestine of the elderly groups significantly increased after CHIKV infection (O-V and O-Abx&V), and cell proliferation levels were also increased (Figure 6(g), Supplementary Fig. S4A). The upregulation of *AREG* and the mucosal repair marker, *TFF3*, in goblet cells (in O-V and O-Abx&V) indicated regeneration of intestinal epithelial and mucosal barrier (Figure 6(h)).

Collectively, CHIKV infection causes a systemic inflammatory burden, primarily promoting inflammation rather than mechanical damage, and activates intestinal IFN signaling to assist gut repair and mucosal regeneration. CHIKV infection caused a more severe inflammatory environment and stronger IFN signaling in the intestines of the elderly group than those in the adult group. However, systemic IFN levels were low, particularly in the Abx-infected elderly group (A-Abx&V), possibly causing more severe pathological damage in the elderly group. Further functional validation using knockout mice is required.

## Discussion

The interaction between the gut microbiota and host reportedly influences immune responses to viral infections.^20^ Murine norovirus infection exhibits beneficial functions and mitigates antibiotic-induced gut damage in mice lacking gut microbiota, highlighting the complex interplay between viral infections, gut immunity, and gut microbiota.^21^ There are several studies indicating that microbiota are involved in the regulation of immune response to alphavirus infections. LPS of skin microorganisms inhibit alphavirus replication by directly interacting with viruses.^22^ GI microbiota shift caused by CHIKV infection is indirectly involved in the modulation of inflammasomes and IL-17 inflammatory cytokine.^19^ Clostridium scindens-derived metabolite can restore pDC- and MyD88-dependent type I IFN responses to restrict systemic CHIKV infection.^23^ Dietary fiber, fermented by GI microbiota, promotes a healthier and more diverse microbiome by providing food for beneficial bacteria and producing beneficial metabolites. However, mice fed with a high-fiber diet show a clear exacerbation of CHIKV arthropathy, with increased edema and neutrophil infiltrate.^24^ Chronic arthritic symptoms caused by CHIKV infection resemble those of RA, further suggesting that CHIKV infection development and progression may be associated with gut immunity and the microbiome.^25^ In this study, we performed a multi-omics integrative analysis of fecal and GI microbiota, microbial metabolism, and host immune responses in the GI tract, spleen, and blood after CHIKV infection based on an established animal model. CHIKV infection drives changes in the structure and metabolism of the gut microbiota, and gut microbiota depletion could enhance CHIKV infection. CHIKV infection imposes a systemic burden on the host, creating a proinflammatory microenvironment in the gut rather than causing direct mechanical damage. Activation of IFN signaling may facilitate gut repair and mucosal regeneration. The elderly group exhibited stronger inflammatory responses, but lower antiviral responses to CHIKV infection compared with that of adult animals.

The use of oral antibiotics to deplete the gut microbiota is common, particularly to avoid immune deficiencies in germ-free mice, such as the underdevelopment of gut-associated lymphoid tissues and immune structures, such as Peyer’s patches and mesenteric lymph nodes.^26^ Notably, innate and adaptive antiviral immune responses are impaired after antibiotic-induced microbiota depletion with increased susceptibility to WNV, DENV, ZIKV, LCMV, and IAV viral infections.^27–29^ Consistently in this study, antibiotic consumption caused higher viral loads and more severe pathological damage in CHIKV-infected animals compared with those of animals without antibiotics treatment, particularly in the elderly group. Furthermore, CHIKV infection caused a significant increase in the abundance of *Prevotella sp*. in fecal samples and intestinal contents. The significant association between abundant *Prevotella sp.*, particularly *Prevotella copri*, and RA and ankylosing spondylitis has been consistently demonstrated through the identification of sequence homology between RA-specific autoantigens (N-acetylglucosamine-6-sulfatase and filamin A) and epitopes (sulfatase proteins) in *P. copri* proteins.^30^ Additionally, we observed a significant increase in the abundance of intestinal *B. fragilis* after infection, particularly in the Abx group, which is a characteristic of the *Prevotella-*dominated gut environment. High abundances of *Prevotella sp*. and *B. fragilis* reportedly modulate the intestinal IFN pathway and T cell involvement in antiviral immunity.^31^ *B. fragilis* primarily interacts with colonic immune cell TLR4-TRIF receptors through its polysaccharide A on the outer membrane, activating the immune protective signaling pathway and promoting the secretion of antiviral IFNs.^32^ Furthermore, *B. fragili* increases dihydrofolate synthesis, which regulates joint inflammation.^33^ Carasso et al. demonstrated that colonization with *B. fragilis* can induce the resolution of colonic inflammation in mice with inflammatory bowel disease.^34^ Similarly, in this study, activation of IFN, the IFN receptor, and ISG after CHIKV infection were particularly prominent in the intestine of the Abx group, whereas the opposite was observed in the blood. The hub genes identified in this study, including *Trim12c*, *Tgtp2*, *Rnf114*, and *Ly6c*, are involved in IFN regulation. *Trim12c* encodes a TRIM5-like protein with ubiquitin ligase activity that interacts with TNFR-associated factor 6, a central regulator of the IFN pathway induced by IFN and pathogen stimulation. Trim12c restricts the replication of human immunodeficiency virus (HIV) and other retroviruses.^35^ RNF114 belongs to the RING domain E3 ubiquitin ligase family and is involved in regulating dsRNA-induced IFN production, NF-κB activity, and T cell activation.^36^ *RNF114* overexpression enhances IRF3 activity and increases types I and III IFN mRNA levels, promoting IFN production.^37^

The IFN alteration post-CHIKV infection in this study was consistent with the published study, which revealed the systemic production of IFN via TLR7-MyD88 signaling. Additionally, Ly6C^hi^ blood monocyte levels significantly increase after antibiotic treatment.^23^ The results in this study implicate that the microbiome modulates the permissiveness of Ly6C^hi^ monocytes to CHIKV in the blood, which affects viremia at the early stages of infection by downregulating IFN and ISG.^23^ However, *Ly6C* expression was upregulated in the blood and intestine after CHIKV infection regardless of antibiotic treatment, with a significant increase in the Abx elderly group that exhibited highly activated intestinal ISGs (Figure 6(b)). Abx treatment attenuates IFN signaling in various viral infections, particularly in non-enteric viruses. Therefore, it may promote viral infections.^38^ The relationship between microbiota and blood IFNs, particularly when the mechanisms of action of antibiotics are partially understood. Therefore, results should be interpreted with caution. Viral infections may cause GI tissue damage and increased permeability, promoting the production of inflammatory cytokines.^20^ CHIKV infection caused no histological damage, but induced an inflammatory environment in the GI tract, particularly in the elderly group. Goblet cells increased after infection in this study.^19^ Mucins are secreted by intestinal stem cell-derived goblet cells at the base of crypts and are crucial in protecting GI tissues from microbial invasion.^39^ Furthermore, owing to the reparative effects of IFN signaling on intestinal epithelial cells, the epidermal growth factor receptor (EGFR) signaling pathway, particularly the IFN-dependent expression of the EGFR ligand AREG, is a critical pathway for intestinal epithelial regeneration and mucosal barrier reconstruction.^40^ In this study, *AREG* and *TFF3*, which are expressed by goblet cells to inhibit apoptosis and promote mucosal recovery, were upregulated after CHIKV infection, particularly in the elderly group, indicating the damage repair of the intestine activated by IFN after CHIKV infection.^41^

We hypothesized that CHIKV infection causes systemic inflammation in the host and IFN signaling and ISG activation are involved in antiviral immune defense. Depletion of the gut microbiota increases the systemic CHIKV burden and downregulates IFN expression in the blood, causing enhanced infection and high viremia. However, the abundance of the gut microbes involved in regulating IFN, such as *B. fragilis* and *Prevotella sp*, is modulated by CHIKV infection to promote intestinal damage repair with host immunity. CHIKV infection induced a more severe inflammatory environment and lower IFN levels in the elderly group, particularly in the O-Abx&V group than those in the adult group, which may be the major reason for the severe pathological damage in the elderly group (Supplementary Fig. S5). Nevertheless, further experimental validations, such as microbial transplantation, are required on the mechanisms of microbiota alterations involved in CHIKV pathogenesis. Primary bile acids are synthesized from cholesterol in the liver, converted into secondary bile acids by anaerobic bacteria in the intestine, and subsequently reabsorbed back to the liver for the production of glycine and taurine.^42^ Microbial-derived (*Clostridium scindens*) and exogenous secondary bile acids promote the systemic type I IFN response and restrict CHIKV infection.^23^ Abx treatment leads to decreased deoxycholic acid (DCA) levels in the mouse intestine, and the exogenous addition of DCA restores the IFN response and reduces viral titers, suggesting that microbial-derived secondary bile acids are critical in limiting CHIKV infection.^23^ Similarly, in this study, intestinal free bile acids significantly decreased after CHIKV infection. Conversely, conjugated bile acids, particularly the conjugates of taurine and bile acids, such as taurocholic, TCDCA, and taurohyocholic acids, were significantly upregulated after CHIKV infection, which was not affected by Abx treatment. Moreover, as an upstream compound of TCDCA and taurocholic acid (TCA), taurine was increased and synchronized with CHIKV infection. The level of plasma TCA was previously closely associated with inflammatory biomarkers in patients with HIV coinfected with hepatitis C virus.^43^ TCA can promote the formation of serum IL-1 and IL-6 by enhancing humoral immunity through the inhibition of the phagocytic activity of monocytes and macrophages.^44^ Furthermore, decreased apoptosis in fibroblast-like synoviocytes (FLS) may be associated with RA activation.^45^ TCDCA significantly increases apoptosis in the FLS of patients with arthritis in a dose-dependent manner.^46^ TCDCA supplementation improves the progression of adjuvant arthritis in rats and attenuates bone destruction.^47^ We speculate that the increased abundance of TCDCA driven by CHIKV infection may indirectly reflect intestinal immune responses. However, because of the lack of knowledge of specific microbes involved in bile acid synthesis, particularly TCDCA and TCA, further experiments, including microbiota transplantation, are required to explore the mechanism by which bile acids influence CHIKV infection-induced arthritic inflammation.

Our findings suggest that CHIKV infection induces systemic inflammation in hosts, which is exacerbated by the depletion of the gut microbiota. However, collaborations between CHIKV-induced gut microbiota alterations and host immunity, modulate intestinal damage repair via microbial metabolites, which is associated with IFN signaling (Supplementary Fig. S5). Nevertheless, this study has two limitations. First, the use of animal models for CHIKV infection may not fully translate to human CHIKV infections. Therefore, clinical samples from CHIKV-infected patients are necessary to validate the findings of this study. Second, regarding the long-term joint inflammation in CHIKV patients, the roles of the gut and its microenvironment in chronic joint inflammation require further investigation to elucidate the “gut-joint” axis during CHIKV infection. Overall, this study offers new insights into the diagnosis, treatment, and development of novel therapeutics for CHIKV.

## Methods

### Statements of animal ethics and biosafety

The Institutional Animal Care and Use Committee of the Institute of Medical Biology, Chinese Academy of Medical Science, approved all animal procedures (approval number: DWSP202207008). All experiments involving live CHIKV were performed in a biosafety level 3 laboratory (BSL-3) or animal BSL-3 laboratory (ABSL-3).

### CHIKV strain and animal experimental procedures

In this study, the CHIKV strain was isolated from the serum sample of a CHIKV RNA-positive patient by the Institute of Medical Biology, Chinese Academy of Medical Sciences, and confirmed as the Asian strain through sequencing (GenBank: MH670649.1).^19^ The mouse-adapted CHIKV strain, after four serial passages in C57BL/6 mice, was amplified in Vero cells for animal infection experiments. A total of 47 C57BL/6 mice were used in this study, including the adult (A) (age, 4–6 weeks) and the elderly (O) (age, 1 year) groups. Each group was randomly divided into three subgroups with different treatments: non-treatment (ANC, *n* = 4; ONC, *n* = 6), CHIKV infection (A-V, *n* = 6; O-V, *n* = 6), antibiotic treatment (A-Abx, *n* = 3; O-Abx, *n* = 6), and CHIKV infection after antibiotic treatment (A-Abx-V, *n* = 8; O – Abx&V, *n* = 8). To avoid cross-contamination and minimize disruptions to rearing conditions for gut microbiota, each mouse was housed separately under pathogen-free conditions and bred with a normal 24-h rhythm. All mice were provided with the same commercial mouse chow and sterile water in an ABSL-3 lab. To eliminate external factors, all mice were acclimatized before the experiment. The animals were injected with CHIKV at 2 × 10^7^ plaque-forming units through the leg muscle (50 μL for each leg), and the NC group was treated with phosphate-buffered saline. Mouse gut microbiota were depleted through treatment with a combination of five antibiotics as follows: 3 mg/mL bacitracin (184862, J&K Scientific), 1.5 mg/mL neomycin (neomycin sulfate, HY-B0470, MCE), 0.8 μg/mL natamycin (HY-B0133, MCE), 0.7 mg/mL meropenem (meropenem Trihydrate, M2279, J&K Scientific), and 0.7 mg/mL vancomycin (vancomycin hydrochloride 122,263, J&K Scientific), followed by a 2-day buffering to exhaust the antibiotics.

According to the pilot experiment, fresh fecal samples were collected for *16S* rRNA-seq at 2 dpi (the acute phase of viremia). All animals were euthanized and dissected at 3 dpi. Intestinal contents were harvested and divided into two parts, each used for metagenomic and metabolomic analyses, respectively. Blood and tissue samples (the small and large intestines, spleen, and ankle) were harvested for further indicated analyses (Figure 1a).

### Determination of viral load in the blood, feces, and other tissues

According to the manufacturer’s instructions, the Direct-zol RNA Miniprep Extraction Kit (Zymo Research, Catalog no. R2052) was used to extract the RNA. The viral load of CHIKV in samples was determined via Taq-Man RT-PCR analysis of the copy number of the viral E1 gene fragment with primers of CHIKV E1F (CTCATACCGCATCCGCATCAG), E1R (ACATTGGCCCCACAATGAATTTG), and the probe CHIKV E1-P (FAM-TCCTTAACTGTGACGGCATGGTCGCC-BHQ1).

### Measurement of footpad thickness and histopathologic analysis

Animals were marked with lines on the forelimb footpads before the infection. Footpad thickness in the same position was recorded daily for 3 consecutive days after treatment. After dissection, all tissue samples, including ankles, were fixed in 10% neutral buffered formalin for at least 10 days to inactivate CHIKV. Parafilm-embedded tissues were sectioned into 3-um sections. The sections were stained with hematoxylin-eosin. Statistical analysis of the pathological damages (inflammatory cell infiltration, eroded ossicles, and tissue bleeding) was conducted using the combined scores of histological lesions (0, absent; 1, slight; 2, mild; 3, moderate; 4, severe) and inflammatory cell infiltration (0, absent; 1, slight; 2, mild; 3, moderate; and 4, severe).

### Immunohistological analysis

Parafilm-embedded tissues were sectioned into 3-μm sections that were then incubated with anti-ZO-1 (Thermo Fisher Scientific, Catalog no. 33–9100; 1:100) and anti-EpCAM (Abcam, Catalog no. ab282457; 1:100) antibodies, followed by the addition of the corresponding fluorescent-conjugated secondary antibodies. The sections were counterstained with DAPI for immunofluorescence analysis. Immunohistochemical staining for CC3 and Ki67 involved incubation of anti-CC3 (Catalog no. GB13436; 1:500) and anti-Ki67 (catalog no. GB111499; 1:400) antibodies, respectively. The Aipathwell software (Service Bio) was used to identify positively stained cells on each slide that was scanned under 3DHISTECH. The results were plotted, and statistical analysis (one-way analysis of variance [ANOVA]) was performed using GraphPad Prism 8.0.

### AB-PAS staining

Goblet cells in the mucosa of the small and large intestines were stained via an AB-PAS staining kit (Catalog no. G1049, Solar Bio) following the manufacturer’s instructions, which was scanned under 3D HISTECH. For goblet cell quantification, each slide was divided evenly into four parts. Followed, five crypts in each part were randomly selected for counting goblet cells. Data were plotted and statistical analysis (a one-way ANOVA) was performed using GraphPad Prism 8.0.

### 16S rRNA-sequencing of the microbial communities in feces

Fecal samples were collected into DNA/RNA Shield^TM^ Lysis Tubes (ZymoBIOMICSTM, Catalog no. R1103–50) at 2 dpi, in which RNA was extracted using a DNA Miniprep Kit (ZymoBIOMICSTM, Catalog no. D4300). Bacterial *16S* rRNA genes (V3–V4) were amplified with primers 338F (5’-ACTCCTACGGGAGGCAGCAG-3’) and 806 R (5’-GGACTACHVGGGTWTCTAAT-3’) in ABI GeneAmp® 9700 PCR thermocycler (ABI, CA, USA). The PCR reaction mixture includes 10 μL 2 × Phanta Master Mix, 0.8 μL forward primer (5 μM), 0.8 μL reverse primer (5 μM), 10 ng of template DNA, and ddH2O to a final volume of 20 µL. The PCR conditions were as follows: initial denaturation at 95 ℃ for 3 min, followed by 29 cycles of denaturation at 95 ℃ for 30 s, annealing at 53 ℃ for 30 s, extension at 72 ℃ for 45 s, single extension at 72 ℃ for 10 min, and cooling at 10 ℃.

After purification of the above PCR product, the final library was constructed and sequenced using Illumina PE300 by Majorbio Bio-Pharm Technology Co, Ltd. (Shanghai, China). Subsequent analysis was conducted using the QIIME2 pipeline [version 2022.11] with the *16S* rRNA 99% packet (V138) from the SILVA database (https://www.arb-silva.de/download/archive/qiime), which classifies self-training (naive-bayes) according to our primer sequences for microbial annotation. The alpha and beta diversities (PCoA) of ASVs were calculated using the Mothur v1.40.5 and Vegan v2.5–3 packages, respectively. Alpha diversity and PCoA were statistically analyzed using the Wilcoxon rank-sum test and permutational analysis of variance (PERMANOVA), respectively.

### Analysis of intestinal microbiome using metagenomic sequencing

Intestinal contents were collected into DNA/RNA ShieldTM Lysis Tubes (ZymoBIOMICSTM, Catalog no. R1103–50), the DNA of which was extracted using a DNA Miniprep Kit (ZymoBIOMICSTM, Catalog no. D4300). To construct paired-end libraries, DNA extracts were fragmented (400 bp) and sequenced using NEXTFLEX Rapid DNA-Seq (BioScientific, Austin, TX, USA). MiSeq sequencing (paired-end, Illumina NovaSeq 6000) was performed by Majorbio Bio-Pharm Technology Co, Ltd. Raw data were trimmed using FASTQ, during which adaptors and low-quality reads (defined as those with < 50 bp, having a quality value below 20, or containing N bases) were eliminated. The host genome was filtered using BWA (http://bio-bwa.sourceforge.net, version 0.7.17) and assembled using MultipleMEGAHIT (https://github.com/voutcn/megahit, version 1.1.2) software. The high-quality reads from each sample were aligned to a non-redundant gene set (with 95% identity) constructed by CD-HIT 4.7 (90% sequence identity, 90% coverage) to determine gene abundance. This alignment was performed using SOAPaligner (version soap2.21release, https://github.com/ShujiaHuang/SOAPaligner). Subsequently, DIAMOND (version 2.0.13, available at http://ab.inf.uni-tuebingen.de/software/diamond/.) was used to align the non-redundant genes with the NCBI NR database for microbial annotation, with an e-value cutoff of × 10^−5^ and selecting the best-hit. Gene functions were annotated from Orthologous Groups and VFDB, and Carbohydrate-Active enzymes were determined for each database. Significantly different (all-against-all) species were screened using LEfSe analysis (http://huttenhower.sph.harvard.edu/galaxy/root?tool_id=lefse_upload). The Kruskal – Wallis H test was used for the statistical comparison among multiple groups (false discovery rate [FDR]; Scheffé 0.95) using R statistics (version 3.5.1). PCoA was performed using PERMANOVA (vegan R package; https://cran.r-project.org/package=vegan). The correlation heatmap was calculated and plotted using the R pheatmap package.

### Analysis of the metabolites in intestinal content via LC-MS

According to the SOP of the ABSL-3 laboratory, virus inactivation in the intestinal contents was achieved using a 1:1 aqueous methanol solution. Samples were then sent to Majorbio Bio-Pharm Technology Co, Ltd. for metabolomic analysis. Briefly, metabolites in the intestinal contents were extracted by grinding 100 mg of the sample with 6 mm grinding beads for 6 min (−10 °C at 50 hz) in methanol: water solution (4:1) containing 0.02 mg/mL L-2-chlorophenylalanine as an internal control. An ACQUITY HSS T3 column (Waters, USA) was used for LC-MS on a Thermo UHPLC-Q Exactive HF-X system under chromatographic and MS conditions. The metabolites were annotated using the HMDB database (http://www. hmdb.ca/), and annotations of anion and cation metabolites were combined during metabolomics analysis. The metabolites in the intestinal contents were associated with the traits identified through WGCNA using the R package (networkType: signed; soft power:12; minModuleSize:30; minKMEtoStay:0.3; MergeCutHeight:0.25) to screen candidate hub metabolites.

### Transcriptomic analysis via Illumina sequencing

Blood, spleen, and intestinal tissues were collected into each 2-mL cryotube containing 500 μL RNAlater. RNA was extracted using the RNeasy Universal Kit (QIAGEN, Catalog no. 73404). High-quality RNA (OD260/280 = 1.8–2.2, OD260/230 ≥ 2.0, RIN ≥ 6.5, 28S:18S ≥ 1.0, >1 μg) was used for further library construction via Illumina NovaSeq Reagent Kit and sequenced using the NovaSeq 6000 system by Majorbio Bio-Pharm Technology Co, Ltd. The raw reads were trimmed, and the quality was controlled using fastp (default parameters) with the default parameters. Subsequently, the clean reads were separately aligned to the reference genome (Musmusculus GRCm39; http://asia.ensembl.org/Mus_musculus/Info/Index) and assembled using HISAT3-N and StringTie (version 2.2.0). Gene expression levels for each transcript were quantified using transcripts per million reads for normalization using RSEM software (version 1.3.3). DEGs (|log2FC|≥2 and FDR ≤ 0.05) were identified using Empirical Analysis of Digital Gene Expression in R (EdgeR). Subsequent functional enrichment analyses of Gene Ontology and Kyoto Encyclopedia of Genes and Genome pathways were conducted using GOATOOLS and KOBAS, respectively, to elucidate gene function and associated pathways. One-way ANOVA (Scheffé 0.95) was used for the statistical comparison of groups using R statistics (version 3.5.1). The blood genes were associated with traits identified using WGCNA with the R package (networkType: signed; soft power:8; minModuleSize:30; minKMEtoStay:0.3; mergeCutHeight:0.25).The candidate hub genes for further network analysis using R (WGCNA igrah).

## Supplementary Material

Sup fig.docx

## Data Availability

Sequence data that support the findings of this study have been deposited in the NCBI database (PRJNA1202677 and PRJNA1206756). Details are provided within the manuscript.

## Abbreviations

CHIKV,: chikungunya virus;
WA,: West African;
ECSA,: East/Central South African;
IOL,: the Indian Ocean lineage;
Treg,: regulatory T cells;
RA,: rheumatoid arthritis;
LPS,: lipopolysaccharides;
MAMP,: microbe-associated molecular pattern;
GI,: gastrointestinal;
NHP,: non-human primate;
ABLS3,: animal biosafety level 3;
PBS,: phosphate-buffered saline;
PFU,: plaque-forming units;
AB-PAS,: Alcian blue and Periodic acid-Schiff;
OTUs,: operational taxonomic units;
H&E,: hematoxylin-eosin staining;
ZO-1,: Zonula occludes protein 1;
EpCAM,: epithelial cell adhesion molecule;
PCoA,: principal coordinate analysis;
PERMANOVA,: permutational analysis of variance;
PLS-DA,: partial least squares discriminant analysis;
db-RDA,: distance-based redundancy analysis;
PICRUSt2,: Phylogenetic Investigation of Communities by Reconstruction of Unobserved States;
VIP,: variable important in projection;
FDR,: false discovery rates;
DEGs,: differentially expressed genes;
GO,: Gene Ontology;
KEGG,: Kyoto Encyclopedia of Genes and Genomes;
WGCNA,: weighted gene co-expression network analysis;
IBD,: Inflammatory Bowel Disease;
TLR,: Toll-like receptor;
