# Supplementary material for: Chikungunya virus drives gut microbiota shifts and IFN-Mediated intestinal repair: insights into microbiota-immune interplay
Source: Gut Microbes. 2025 Jun 5;17(1):2512900. doi: 10.1080/19490976.2025.2512900 (PMC12143707; doi:10.1080/19490976.2025.2512900)
Supplement: Sup fig.docx [file KGMI_A_2512900_SM7483.docx]

**
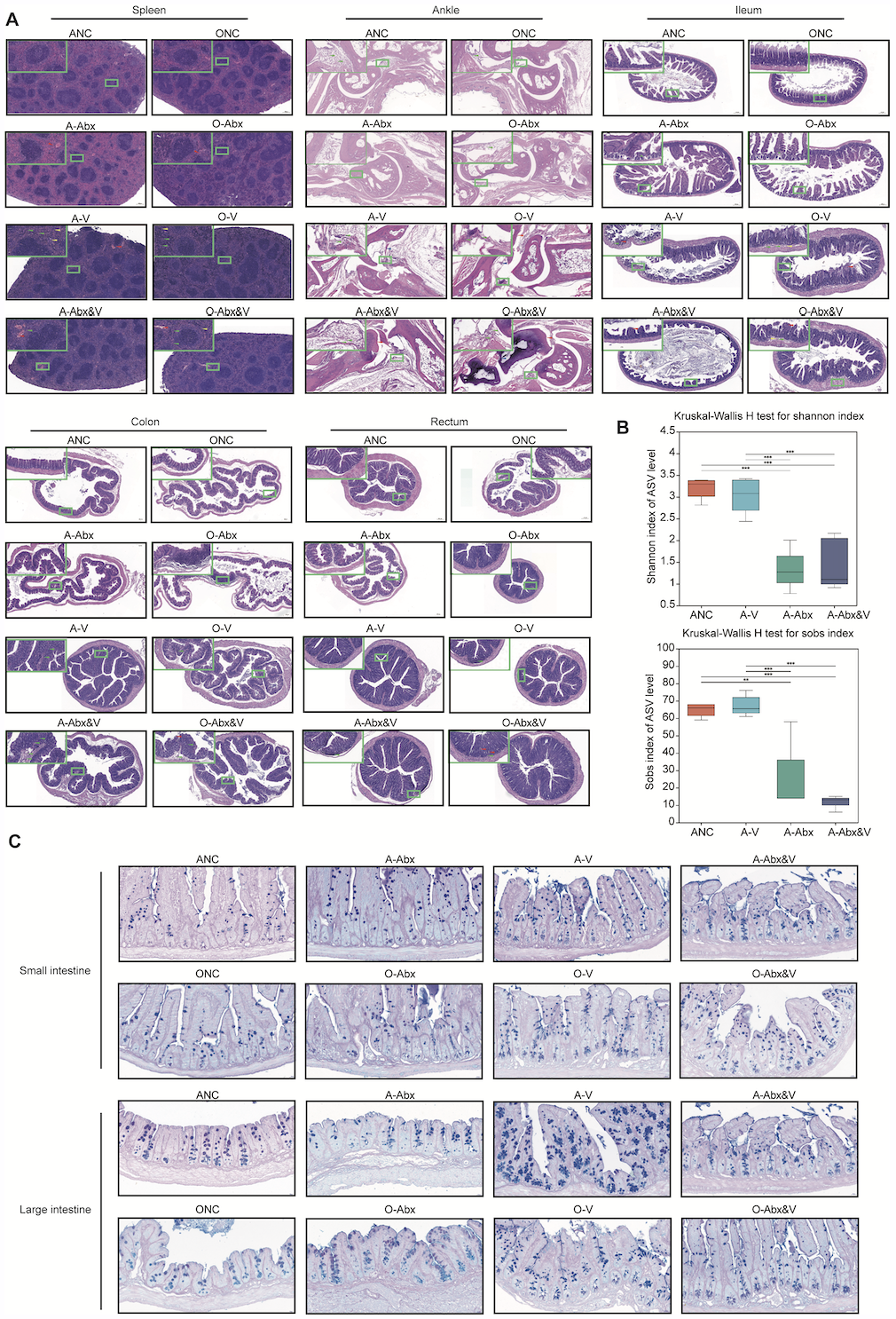
Supplementary Figure 1 Histopathological analysis of GI and other tissues**

(**A)** Related to Fig 1D，H&E staining of spleen, ankle and GI tissue sections. At 3 dpi, the animals were euthanized and dissected. The indicated tissues were harvested and processed for H&E staining and histopathological evaluation as described in the section Methods. The scale bars are 20 μm.

**(B)** Alpha diversity of fecal microbiota. Shannon index in each group of fecal microbes (Top), andSobs index in each group of fecal microbes (Bottom). *Wilcoxon rank-sum test* and post hoc *Scheffe* test, FDR corrected; ** p < 0.01).

**(C)** Related to Fig 5f, representative images of mucin goblet cells by the AB-PAS staining. The scale bars are 20 μm.

**
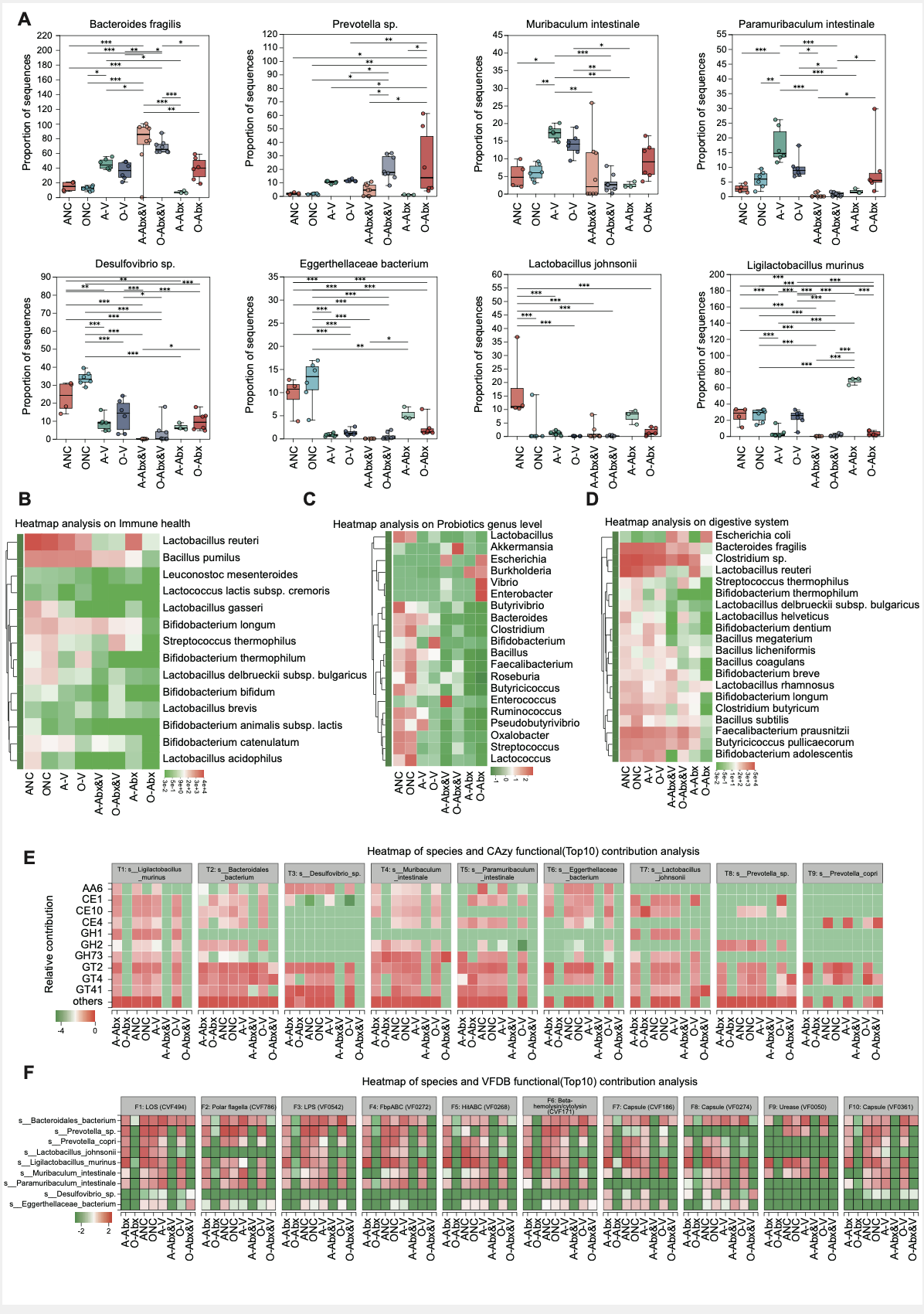
Supplementary Figure 2, related to Fig. 2.**

**(A)** Comparative statistical analysis of abundance of key microbes (identified by LEfSe) between each groups. ( *Wilcoxon rank-sum test*; and post hoc *Scheffe* test, FDR corrected; *p < 0.05, ** p < 0.01, *** p <0 .005).

**(B)** Heatmap of the abundance of microbiota related to immune responses in each group.

**(C)** Heatmap of the abundance of probiotics (genus level) in each group.

**(D)** Heatmap of the abundance of microbiota related to GI functions in each group.

**(E)** Heatmap of the relative abundances of virulence factors and enzyme functions **(f)** of microbes in the GI contents of each group. The relative abundance is indicated by the color scale bar on the left.

**
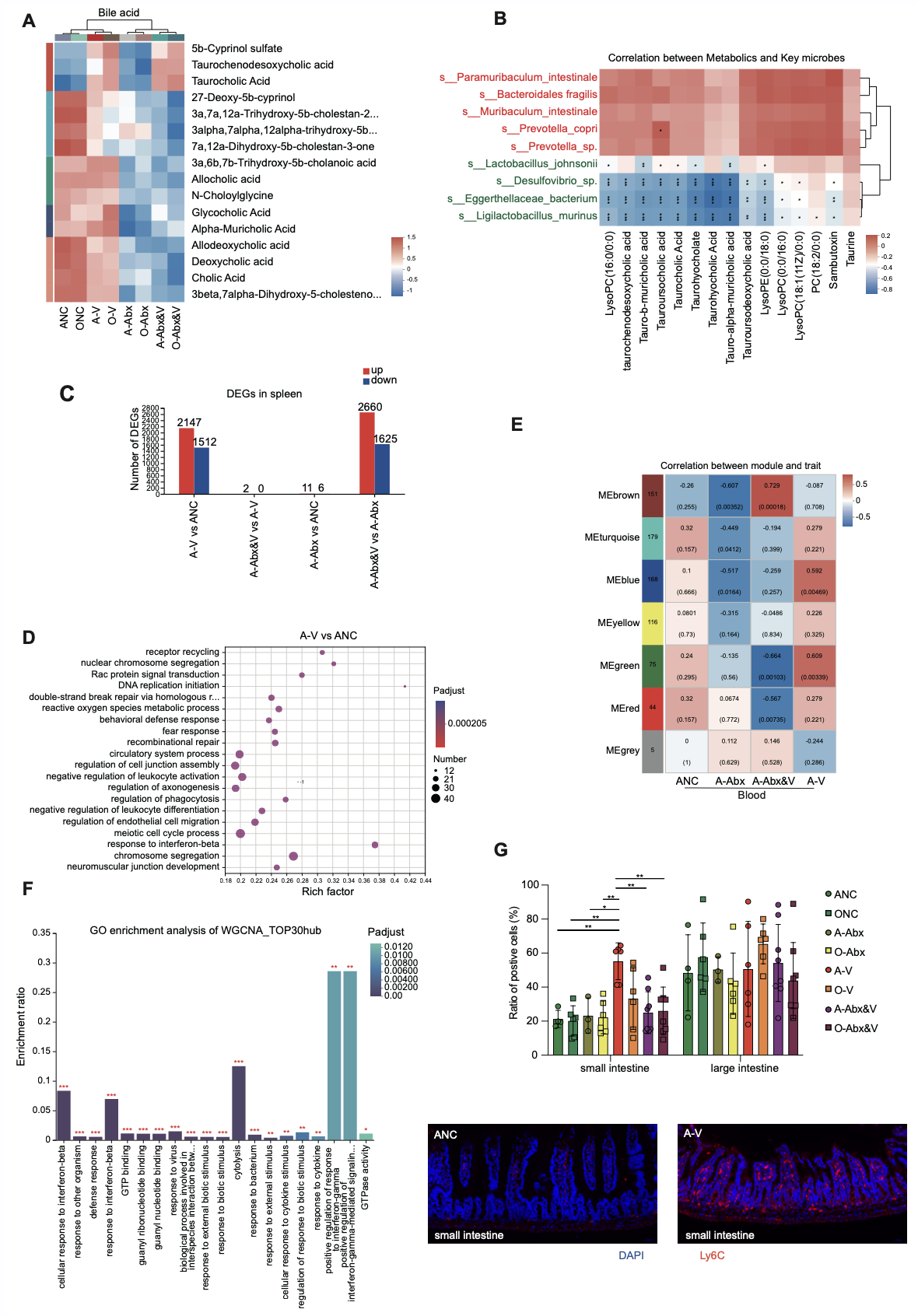
Supplementary Figure 3 Related to Fig 3.**

**(A)** Heatmap of bile acid abundance in intestinal contents.

**(B)** Matrix correlation analysis between key microbiota with conjugates of taurine and bile acids. Red species, upregulated abundance post-infection; Green species, downregulated abundance post-infection.

**(C)** Number of differentially expression genes in spleen of adult group.

**(D)** Functional enrichment analysis of the DEGs in the spleen (A-V vs ANC). The size of the circle indicates the amount of DEGs enriched to corresponding function. Levels of *p* value were expressed by color density as shown in the scale bar.

**(E)** Weighted gene co-expression network analysis (WGCNA) was performed to identify the genes in blood and evaluate their correlations among groups to identify hub genes. The numbers in the left column (modules) indicate the number of genes The numbers in the middle box indicate the correlation coefficients and *p*-values (in parentheses) between the modules and corresponding group. Correlation levels were differentiated by the color of scale bar (red for positive correlation and blue for negative correlation).

**(F)** Functional enrichment analysis of the hub-genes positively correlated with CHIKV infection.

**(G)** Immunofluorescence staining of the Ly6c. Ratio of positive cells was calculated as described in the Methods (top). Representative images were shown here (bottom). *(*One-way ANOVA*; *p < 0.05, ** p < 0.01).

**
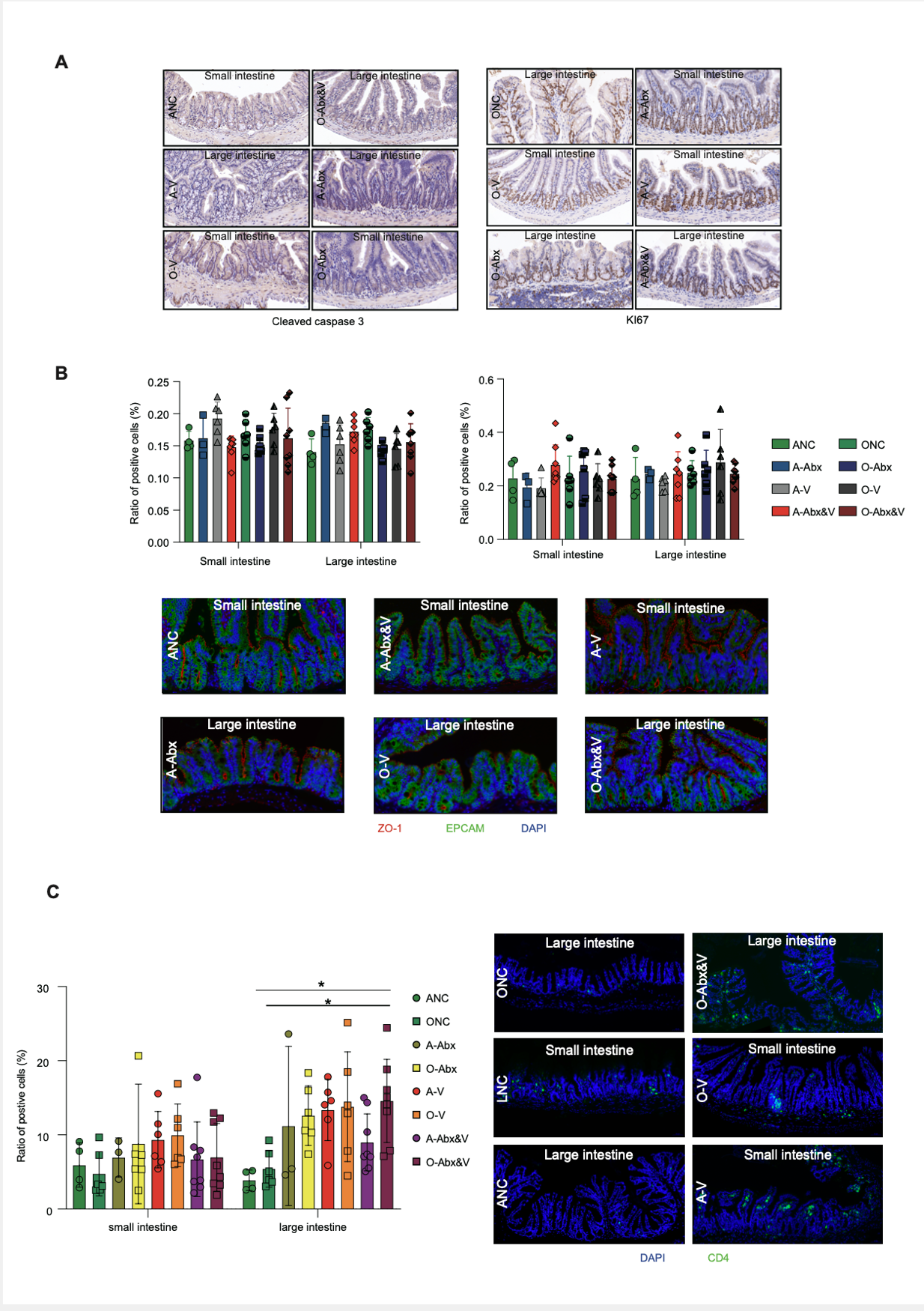
Supplementary Figure 4 Histological analysis of GI tissues**

**(A)** Related to Fig. 5f , representative images of IHC staining of Ki67 (right) and cleaved caspase 3 (left). The scale bars are 20 μm.

**(B)** The epithelial integrity of the GI tract was determined via IHC staining of Zonula occludens protein (ZO-1)(top left) and epithelial cell adhesion molecule (EpCAM)(top right). Ratio of positive cells was calculated as described in Methods (no sigficant differece among groups, *One-way ANOVA*). Representative images were shown here (bottom).

**(C)** Immunofluorescence staining analysis of CD4+ cells in GI tissues. Left, percentage of CD4+ cells. Right, representative images of immunofluorescence staining of CD4+ cells (*One-way ANOVA*; *p < 0.05).

**Supplementary Figure**
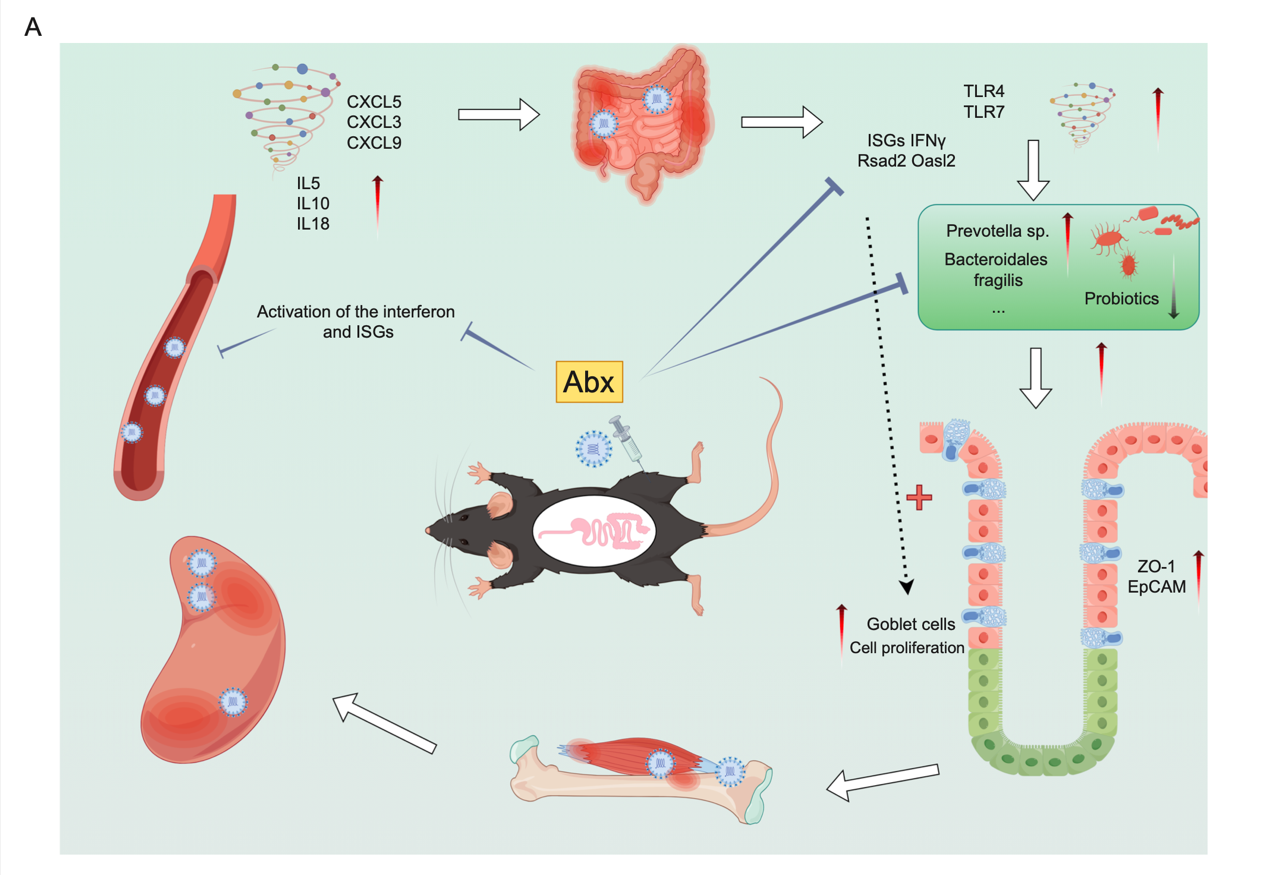
**5** Summary of this study. This figure was prepared via Figdraw (Copyright NO. RWSUY70770)
